# Anti-Fas mAb-induced apoptosis and cytolysis of airway tissue eosinophils aggravates rather than resolves established inflammation

**DOI:** 10.1186/1465-9921-6-90

**Published:** 2005-08-08

**Authors:** Lena Uller, Kristina Rydell-Törmänen, Carl GA Persson, Jonas S Erjefält

**Affiliations:** 1 Dept. Experimental Medical Science Lund University, BMC F10, 221 84, Lund, Sweden; 2 Dept. Clinical Pharmacology Lund University Hospital, Lund Sweden

**Keywords:** asthma, allergy, eosinophils, apoptosis, chemokines

## Abstract

**Background:**

Fas receptor-mediated eosinophil apoptosis is currently forwarded as a mechanism resolving asthma-like inflammation. This view is based on observations *in vitro *and in airway lumen with unknown translatability to airway tissues *in vivo*. In fact, apoptotic eosinophils have not been detected in human diseased airway tissues whereas cytolytic eosinophils abound and constitute a major mode of degranulation of these cells. Also, Fas receptor stimulation may bypass the apoptotic pathway and directly evoke cytolysis of non-apoptotic cells. We thus hypothesized that effects of anti-Fas mAb *in vivo *may include both apoptosis and cytolysis of eosinophils and, hence, that established eosinophilic inflammation may not resolve by this treatment.

**Methods:**

Weeklong daily allergen challenges of sensitized mice were followed by airway administration of anti-Fas mAb. BAL was performed and airway-pulmonary tissues were examined using light and electron microscopy. Lung tissue analysis for CC-chemokines, apoptosis, mucus production and plasma exudation (fibrinogen) were performed.

**Results:**

Anti-Fas mAb evoked apoptosis of 28% and cytolysis of 4% of eosinophils present in allergen-challenged airway tissues. Furthermore, a majority of the apoptotic eosinophils remained unengulfed and eventually exhibited secondary necrosis. A striking histopathology far beyond the allergic inflammation developed and included degranulated eosinophils, neutrophilia, epithelial derangement, plasma exudation, mucus-plasma plugs, and inducement of 6 CC-chemokines. In animals without eosinophilia anti-Fas evoked no inflammatory response.

**Conclusion:**

An efficient inducer of eosinophil apoptosis in airway tissues *in vivo*, anti-Fas mAb evoked unprecedented asthma-like inflammation in mouse allergic airways. This outcome may partly reflect the ability of anti-Fas to evoke direct cytolysis of non-apoptotic eosinophils in airway tissues. Additionally, since most apoptotic tissue eosinophils progressed into the pro-inflammatory cellular fate of secondary necrosis this may also explain the aggravated inflammation. Our data indicate that Fas receptor mediated eosinophil apoptosis in airway tissues *in vivo *may cause severe disease exacerbation due to direct cytolysis and secondary necrosis of eosinophils.

## Background

Apoptosis of inflammatory cells followed by their swift removal through phagocytosis is considered a major mechanism of resolution of inflammatory conditions [1,2]. The most common chronic inflammatory disease, asthma is characterized by eosinophilia, epithelial derangement, plasma exudation, and hypersecretion [3,4]. The role of the eosinophil in this disease is currently under intense investigation [5] and much interest has been devoted to apoptosis of eosinophil granulocytes [6,7]. In the absence of growth factors or in the presence of glucocorticoids, eosinophils *in vitro *exhibit massive apoptosis and, eventually, secondary necrosis [8-10] occurs. A specific mode of inducing death through apoptosis is stimulation of Fas antigen (Fas), a cell surface protein expressed in most cells including eosinophil granulocytes [11]. Fas may also trigger an alternative death pathway leading to cytolysis of cells without prior apoptosis [12]. Eosinophil cytolysis causing extra-cellular spilling of eosinophil granules commonly occurs in asthmatic bronchi [13] but it is not known whether stimulation of the Fas-receptor may evoke cytolysis of eosinophils.

Apoptosis of eosinophil granulocytes is effectively induced *in vitro *by cross-linking of Fas membrane receptors with agonistic anti-Fas monoclonal antibody (mAb) [11,14,15]. Similarly, administration of anti-Fas mAb intra-nasally to the lungs of allergic mice has been shown to induce apoptosis of eosinophils in the airway lumen [7]. This latter finding is of interest because apoptotic eosinophils have also been observed in asthmatic sputa following disease exacerbation [16]. As a corollary it has been suggested that agents inducing eosinophil apoptosis may be developed as novel anti-asthma drugs [17-19]. However, the occurrence of apoptotic cells in the airway lumen cannot tell about the presence of such cells in the airway tissues [20]. Indeed, apoptotic eosinophils have so far rarely have been seen in airway tissues [20] where eosinophils instead may be silently eliminated from the tissue through alternative clearance mechanisms such as egression into the airway lumen followed by mucociliary clearance [21,22]. Even at resolution of established airway eosinophilia, spontaneously or by effects of anti-inflammatory steroids, apoptotic eosinophils have not been detected in lung tissues [21]. The absence of apoptotic eosinophils in human diseased tissues together with the common occurrence of cytolytic eosinophils suggest that these cells are more prone to undergo cytolysis than apoptosis in inflamed airways (35). Also, since inducement of apoptosis in tissue eosinophils has not yet been compellingly demonstrated it remains speculative what actually may result *in vivo *when apoptosis of these cells occurs.

Differing from the prior reports, that focused on airway lumen data [7,23], this study explores airway tissue effects of anti-Fas mAb given to mouse allergic airways with already established eosinophilic inflammation. Importantly, we have included a detailed transmission electron microscopy analysis to assess cell phenotypes such as apoptotic and cytolytic cells that are basically defined by ultrastructural characteristics [24]. Here we demonstrate that anti-Fas mAb evoked apoptosis of more than 1/4th of the airway tissue eosinophils and that a majority of these cells proceeded to undergo secondary necrosis. Direct cytolysis of non-apoptotic tissue eosinophils was also induced by the present anti-Fas mAb treatment. Furthermore, at variance with previous interpretations of findings in the airway lumen and *in vitro *[7,17,23] we now demonstrate that the established allergic inflammation of airway-lung tissues was not resolved. On the contrary, as indicated by a wide range of indices, the allergic eosinophilic inflammation was greatly aggravated producing for the first time in mouse models several hallmarks of human asthma. This *in vivo *study thus demonstrates an unprecedented asthma-like histopathology in mouse airways and, unravels significant risks involved in drug-induced stimulation of death-receptors.

## Methods

### Animals

8–10 weeks old male C57BL/6 mice (Bomholtgard, Denmark) were used. Mice were kept in well-controlled animal housing facilities and fed *ad libitum*. The study was approved by the Regional Ethics Committee in Malmoe-Lund, Sweden.

### Allergen sensitization and challenge protocol

**Figure 1:**
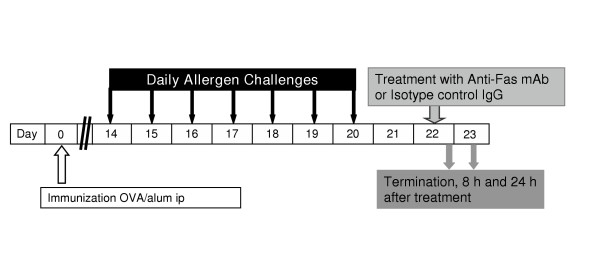
Study design. All animals were immunized with OVA and 14 days later exposed to aerosol challenge with OVA for 7 days to establish tissue and lumen eosinophilia. Treatment with anti-Fas mAb or isotype control IgG was administered intra-nasally at day 22 and outcome measurements including BAL and tissue sampling were made at 8 and 24 h after treatment.

The ovalbumin sensitization and challenge protocol was similar to that described previously [25,26]. Briefly, all mice were immunized to chicken OVA (Grade III; Sigma, St Louis, MO) via i.p injection with 10 μg OVA, adsorbed to 1 mg of alum. Fourteen days after the immunization mice were exposed daily for seven days to aerosolized OVA at a concentration of 1 % wt / vol for 30 minutes. Control animals received saline challenge (Figure 1).

### Study design

After the lung tissue eosinophilia was established (day 22), 30 μg of anti-Fas mAb (purified hamster Anti-mouse Fas antibody, clone Jo2; Pharmingen, Palo Alto, CA) or a matched isotype control antibody (hamster IgG, control) was administered to the lungs via the intranasal route as previously described [7]. The dose of anti-Fas mAb was chosen according to a dose-response study carried out by Tsuyuki et al where 30 μg was markedly effective at inducing apoptosis of airway luminal eosinophils [7]. Unless otherwise stated each experimental group consisted of 8 animals. Outcome measurements were made at 8 and 24 h after each treatment (OVA/OVA+ IgG and OVA/OVA + anti-Fas mAb) (Figure 1). One group of animals with established eosinophilia treated with anti-Fas mAb was followed for 72 h after intra-nasal treatment (n = 3). Importantly, effects of anti-Fas mAb were also examined in mice lacking eosinophilic airway inflammation. Thus mice immunized with OVA and subjected to saline challenges were given anti-Fas mAb (or the isotype control IgG) and airway histopathology examined after 24 h. All animals were sacrificed by ip injection of pentobarbital immediately followed by bronchoalveolar lavage (BAL) and dissection of the lungs and tracheobronchial airways.

### Bronchoalveolar lavage (BAL) and quantification of luminal cells

BAL was performed via a ligated tracheal cannula. One ml of PBS was allowed to passively enter the lungs at a pressure of 10 cm H_2_0. This procedure was carried out twice. The obtained BAL-fluid (BALF) from each animal was immediately centrifuged (700 g, 5 min) and the supernatant frozen for ELISA analysis. The cell pellet was washed and resuspended in 250 μl PBS containing 10% FCS. The total number of cells was quantified using a hemocytometer and 5 × 10^5 ^cells cytocentrifuged to microscope slides. Differential cell counts were performed on May-Grünwald Giemsa stained slides and percentage of eosinophils, lymphocytes, neutrophils, and macrophages determined by counting 200 cells in a blinded manner. To obtain the absolute number of each leukocyte subtype in each BALF, the percentage of cells was multiplied by the total number of cells recovered from the BAL.

### Lung tissue processing for histology analysis

From each animal 4 tissue samples were taken from the superior lung lobes at the level just below the root of the lung. One tissue sample was immersed in Stefanini's fixative (2% paraformaldehyde and 0.2% picric acid in 0.1 M phosphate buffer pH 7.2) overnight, rinsed repeatedly in Tyrode buffer supplemented with 10% sucrose, and finally frozen in TissueTEK (Miles, Inc., Elkhart, IN). The frozen specimens were stored at -80°C until used for histochemistry. A separate sample was immersed overnight in buffered 4% paraformaldehyde (pH 7.2) and thereafter dehydrated and embedded in paraffin. An additional sample was placed in a fixative consisting of a mixture of 3% formaldehyde and 1% glutaraldehyde in 0.1 M phosphate buffer, pH 7.2 and used for transmission electron microscopic (TEM) analysis. The rest of the lung tissue was immediately frozen for mRNA analysis.

### Staining and counting lung tissue eosinophils

Eosinophils were detected by histochemical visualization of cyanide-resistant eosinophil peroxidase (EPO) activity [27]. In brief, 5 μm cryosections were incubated for 8 min at room temperature in PBS buffer (pH 7.4) supplemented with 3.3-diaminobenzidine tetrahydrochloride (60 mg / 100 ml; SIGMA), 30% H_2_0_2 _(0.3 ml / 100 ml), and NaCN (120 mg / 100 ml). Slides were then rinsed in tap water and mounted in Kaisers medium (Merck, Darmstadt, Germany). Eosinophils were identified by their dark brown reaction product and quantified as number of peribronchial eosinophils / 0.1 mm^2 ^tissue area.

### Staining of mucus-containing cells, mucus secretions, and mucus-plasma plugs

5 μm sections of paraffin embedded lung tissue were cut, dewaxed to water and then stained with periodic acid-Schiff reagent (PAS) as previously described [26]. Epithelial integrity was examined and specific signs of injury-repair processes [28] were looked for. A mucus plug index was established as number of large and medium airways with tethered secretions/plugs divided by the total number of airways in each tissue section and multiplied by 100 to obtain percentage values. The presence of mucus plugs was also confirmed by transmission electron microscopy. Immunostaining for fibrinogen was performed using a polyclonal Ab (rabbit anti-fibrinogen 1:320, Dako, Copenhagen Denmark) and visualized using a secondary FITC antibody (swine anti-rabbit 1:80, Dako, Copenhagen, Denmark.).

### Detection of apoptosis, secondary necrosis and eosinophil cytolysis

Apoptotic cells in the lung tissue were mainly detected using TUNEL-technique. A combined staining with TUNEL and Chromotrope-2R identified apoptotic eosinophils as previously described [10]. Importantly, to assess an apoptotic morphology, detect engulfed eosinophils, and different activation grades of eosinophils, ultrastructural analysis using transmission electron microscopy was also performed as previously described [10]. Ultrathin sections (60–80 nm) for electron microscopy were cut on an LKB MK III ultratome and contrasted with uranyl acetate and lead citrate. The sections were examined using a Philips CM-10 transmission electron microscope and the ultrastructural criteria for eosinophil apoptosis were eosinophils displaying cell shrinkage, intact cell membrane and nuclear chromatin condensation as previously described [10]. Secondary necrosis was defined as cells exhibiting typical features of apoptosis e.g. nuclear condensation, but with clear signs of membrane rupture and extra cellular distribution of cell debris. Macrophages were identified using TEM and their content of eosinophil granules or whole eosinophil cell material was also quantified using TEM. Eosinophil cytolysis, which emerges as a major mode of eosinophil degranulation in asthma and rhinitis is characterized by chromatolysis of the cell nucleus and rupture of the cell membrane, whereby the protein-rich specific eosinophil granules are released into the tissue [29].

### Measurement of mRNA expression

Total RNA from the lungs was extracted with RNAzol B (Tel-Test, Inc., Friendswood, TX) according to the manufacturer's protocol. Chemokine mRNA expression was determined by multiprobe RNAse protection assay (RPA) using the Riboquant RPA kit (mCK-5, Pharmingen, San Diego, CA), according to the supplier instructions and as previously described [30]. The identity and quantity of each mRNA species in the original RNA sample were then determined based on the signal intensities given by the appropriately sized, protected probe fragment bands. 1 μg RNA was loaded for each sample and the differences in sample loading were normalized by a factor of the ratio of the housekeeping genes L32 and GAPDH.

### Data Analysis

Histology analyses were performed and quantified in a blinded manner. Tissue sections from eight animals were investigated in each treatment and control group. An Olympus BX60 microscope, equipped with an Olympus DP50 digital camera was used for imaging. Wilcoxon Rangsumtest for statistical analysis was performed using Analyze It™ (Analyse-it software, Ltd. Leeds, UK). Data are expressed as mean ± SEM. A value of p < 0.05 was considered statistically significant.

## Results

### Fas-induced apoptosis and reduced number of eosinophils in the airway lumen

**Figure 2:**
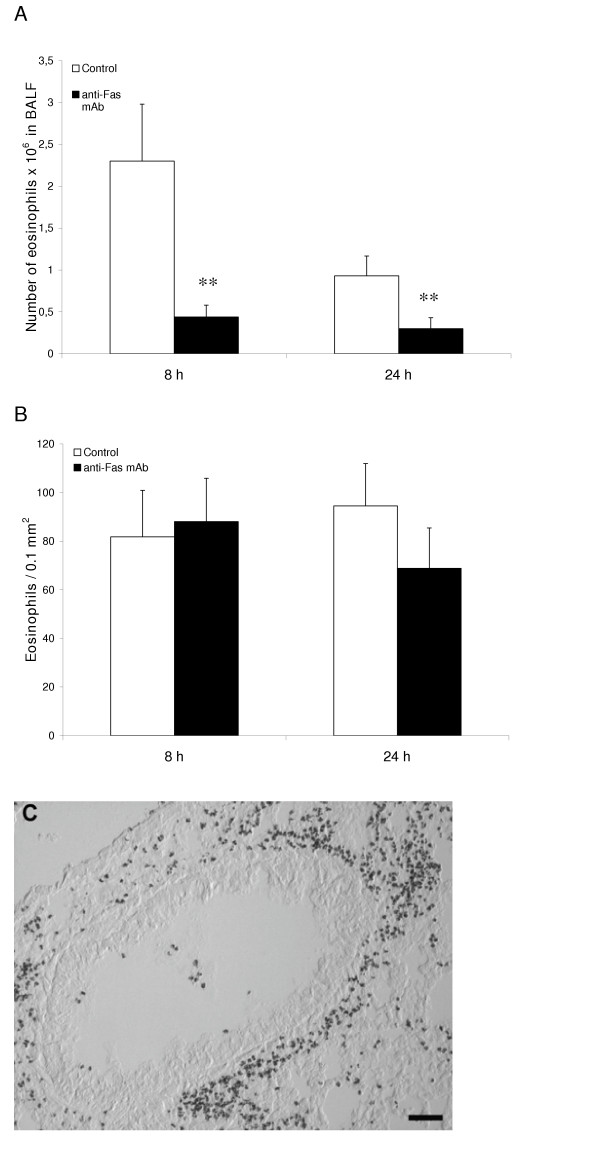
After eosinophilia had been established in the immunized mice anti-Fas mAb or isotype control Ab was administered locally to the lungs followed by BAL and tissue sampling at 8 and 24 hours. The number of eosinophils in airway lumen (A) and airway tissue (B) was quantified as described in detail in the methods section. White bars represent mice given control Ab and black bars mice given anti-Fas mAb. Error bars indicate the standard error of the mean for each group of mice (n = 8, ** = p < 0.01). Peribronchial eosinophilia induced by the OVA challenges is shown in (C).

The present weeklong, daily allergen challenges with OVA (see study design, Figure 1) established a marked airway tissue and lumen eosinophilia. Post-challenge intra-nasal administration of anti-Fas mAb to the lungs of these mice decreased the number of eosinophils in the airway lumen (BALF) at 8 and 24 hours (Figure 2A) compared to animals receiving isotype control Ab. Microscopic analyses of cytospin slides showed that a majority of the lumen eosinophils in anti-Fas treated animals had an apoptotic morphology. Other luminal cells including neutrophils, lymphocytes and monocytes/macrophages remained viable after the anti-Fas treatment. These lumen data agree with previously reported observations [7].

### Fas-induced apoptosis of lung tissue eosinophils and insufficient clearance of apoptotic eosinophils

**Figure 3:**
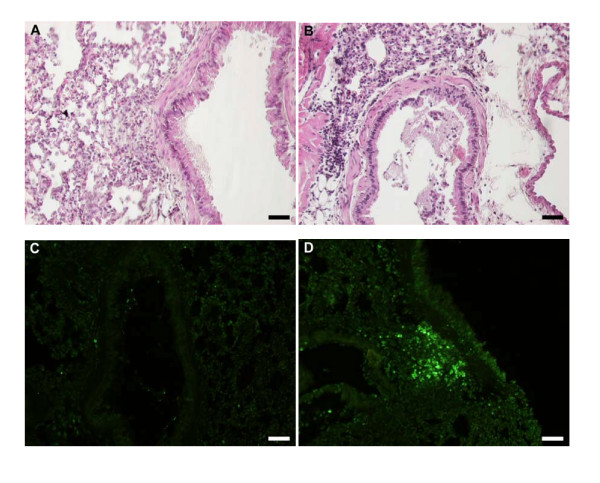
Representative light micrographs of mouse lung tissue using Htx-staining in control (A) and anti-Fas mAb treated animals (B) at 24 h. Htx-staining shows dark condensed (pycnotic) nuclei of eosinophils and disturbed epithelial lining. Very few TUNEL-positive apoptotic cells were present in control treated animals (C) whereas a large number of TUNEL-stained cells was detected in anti-Fas mAb treated animals (D), almost all of which were shown to be apoptotic eosinophils by double chromotrope 2R and TUNEL staining (see also Figure 4).

**Figure 4:**
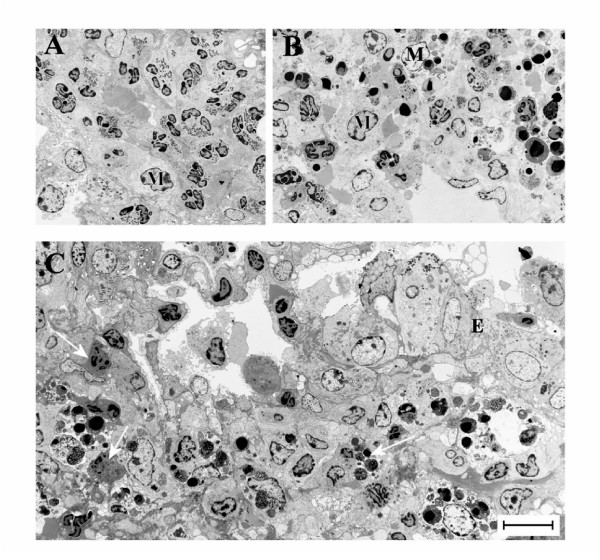
Transmission electron micrographs of lung tissue from mice with OVA-induced eosinophilia. Control mice treated with the isotype control Ab showed no sign of eosinophil apoptosis at 8 h (A) and 24 h (not shown). In mice treated with anti-Fas mAb there were numerous apoptotic eosinophils in the lung tissues at both 8 and 24 hours after treatment (B and C, respectively). The apoptotic eosinophils were rarely engulfed although macrophages (labeled M) commonly occurred in the tissue (B). By 24 h a majority of the apoptotic eosinophils exhibited signs of secondary necrosis and severe inflammation was recorded including neutrophil infiltration (arrow) and derangement of the epithelial lining (labeled E).

**Figure 5:**
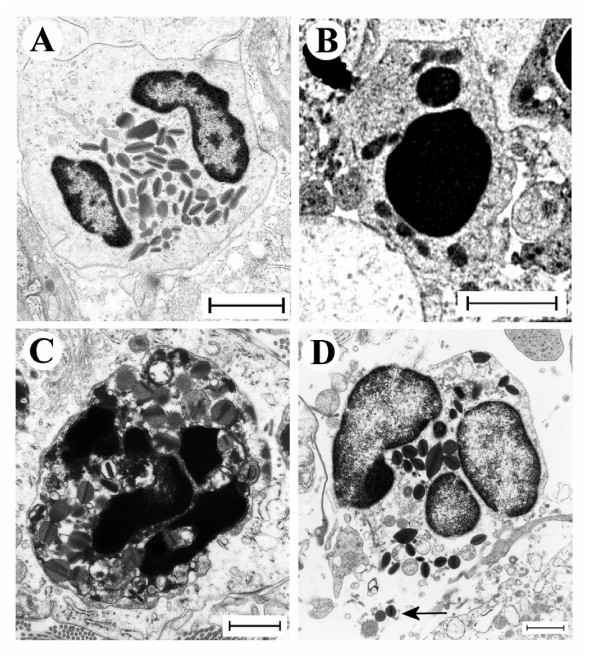
These micrographs illustrate characteristic eosinophil phenotypes present in mouse airways in this study: (A) viable non-degranulating eosinophil, the only phenotype found in lung tissues of allergen challenged animals; (B) apoptotic eosinophil exhibiting nuclear condensation, cell shrinkage, and an intact cell membrane; (C) an apoptotic eosinophil exhibiting secondary necrosis involving cell membrane rupture and piecemeal degranulation; (D) a cytolytic eosinophil exhibiting chromatolysis, cell membrane rupture, and spilling of electron-dense specific granules into the tissue (arrow).

The airway tissue eosinophilia was not reduced by anti-Fas mAb treatment (Figure 2B, and 2C). Yet, contrasting the lack of apoptotic eosinophils in the airway tissues of animals receiving allergen challenge or allergen challenge plus isotype control Ab (Figure 3A, and 3C; Figure 4A; Table 1), apoptotic eosinophils occurred frequently in anti-Fas treated lung tissues especially in granulomas around bronchi and bronchioles (Figure 3B, and 3D; Figure 4B, and 4C; Table 1). Apoptosis is defined by ultrastructural criteria [24]. In this study apoptotic eosinophils were thus assessed not only by staining techniques but foremost by transmission electron microscopy (TEM) analysis demonstrating transformation of the bi-lobular nuclei of normal eosinophils into a condensed dark nucleus and by cell shrinkage occurring without rupture of the cell membrane (Figures 4 and 5). The inability of anti-Fas mAb treatment to resolve the tissue eosinophilia was associated with poor clearance of the tissue eosinophils. As suggested by the reduced lumen eosinophilia (Figure 2A), clearance through egression of cells into the lumen was reduced. There was further an insufficient clearance of apoptotic tissue eosinophils through engulfment (Figure 4B). Indeed, many apoptotic tissue eosinophils underwent secondary necrosis (see below).

**Table 1:** Viable, apoptotic, necrotic and cytolytic eosinophils in mice with OVA-induced established lung tissue eosinophilia.

	Viable eosinophils ^A^	Apoptotic eosinophils^A^		Cytolytic eosinphils^A^	Neutrophils^B^
				
		Intact	Necrotic		
OVA/control 8 h	100	0	0	0	3 ± 1
OVA/FAS 8 h	69 ± 7	19 ± 5	8 ± 3	4 ± 1	12 ± 4
OVA/control 24 h	100	0	0	0	5 ± 0.5
OVA/FAS 24 h	70 ± 4	13 ± 3	15 ± 2	2 ± 0.7	24 ± 2

^A ^= Eosinophil phenotypes are presented as % of total number of eosinophils in each transmission electron microscopy grid (n = 8).

^B ^= Number of neutrophils occurring in each grid (n = 8).

### Fas-induced secondary necrosis of apoptotic tissue eosinophils

Apoptotic eosinophils in the late stages of apoptosis, not being engulfed, proceeded to undergo secondary necrosis. Already at 8 h following treatment with anti-Fas almost half of the apoptotic eosinophils and at 24 h a majority of them exhibited secondary necrosis (Table 1). Typical signs of the secondary necrosis were a condensed dark nucleus and cell membrane rupture (Figures 4C and 5C) as previously described [10]. These cells further exhibited piecemeal degranulation of the specific granules (Figure 5C).

### Primary cytolysis of non-apoptotic tissue eosinophils

After treatment with anti-Fas mAb 2–4 % of the tissue eosinophils exhibited primary cytolysis more so at 8 h than at 24 h (Table 1). As described previously for eosinophils in human diseased airway tissues [13,20,29] these cells were without signs of apoptosis, exhibited little piecemeal degranulation and were characterized by chromatolysis and cell membrane rupture including the spilling of electron dense (protein-rich) specific granules into the tissue (Figure 5D, Table 1).

### Anti-Fas mAb caused up-regulation of CC-chemokines

**Figure 6:**
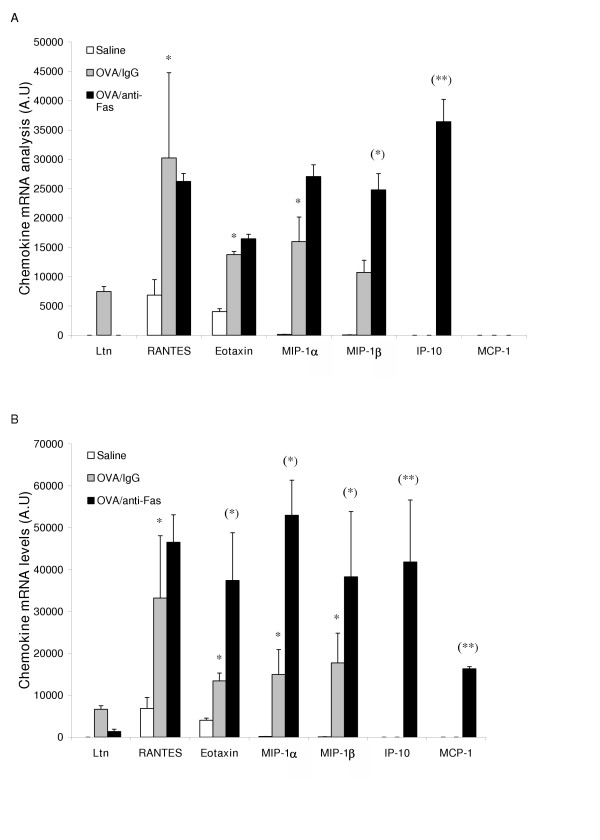
CC-chemokine mRNA expression 8 h (A) and 24 h (B) after treatment with anti-Fas mAb or isotype control (IgG). Two days post allergen challenge expression of 5 different CC-chemokines in the lung was up-regulated compared to immunized control animals receiving saline challenges. Treatment with anti-Fas mAb post allergen challenge further increased the expression of eotaxin MIP-1α, and MIP -1β, and additionally induced the expression of IP-10 and MCP-1. Data are mean ± SEM. **p < 0.01 indicates differences between OVA and saline treatments. §§ p < 0.01 indicates difference between anti-Fas mAb treated and control-treated OVA-challenged animals.

Treatment with anti-Fas mAb resulted in a marked up-regulation of a range of CC-chemokines involved in recruitment of eosinophils and neutrophils. Thus, mRNA levels for MIP-1α, eotaxin, and MIP-1β were up-regulated (Figure 6A, and 6B). Two chemokines, IP10 and MCP-1, that are involved in severe inflammatory processes [31], were not expressed in isotype IgG treated animals, but were induced by anti-Fas mAb treatment (Figure 6A, and 6B).

### Additional signs of Fas-induced aggravation of airway inflammation

**Figure 7:**
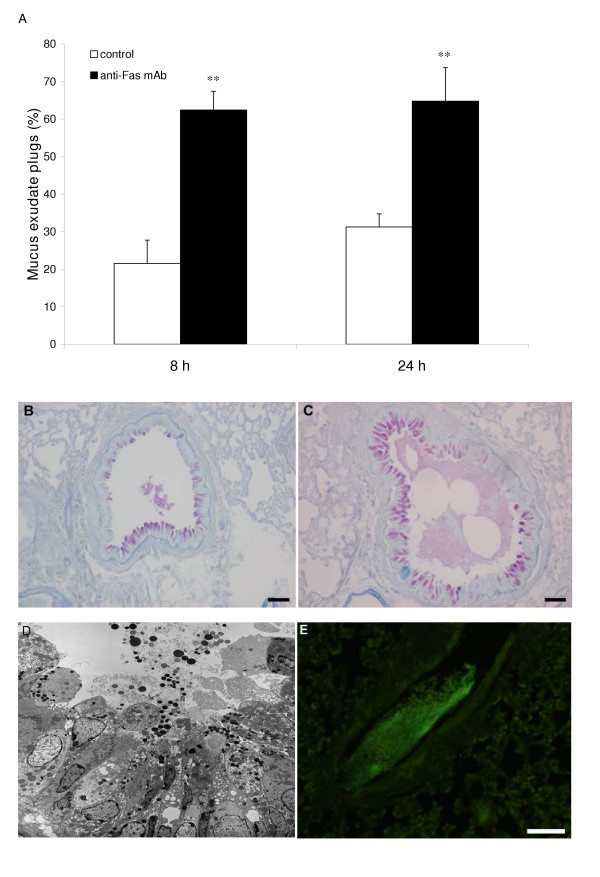
Observations in allergen challenged animals demonstrating anti-Fas-induced mucus-exudate plugs (A,C,D,E) compared to control isotype antibody (IgG) treatment (A,B). The occurrence of mucus-exudate plugs is expressed as percentage affected airways in each tissue section in control mice (white bars) and in mice treated with anti-Fas mAb (black bars) at 8 and 24 hours (A). Bars indicate the standard error of the mean for each group of animals (n = 8, ** = p < 0.01). Histochemical staining with periodic acid-Schiff reagent (PAS) illustrated mucus-containing cells (B,C) and so did the transmission electron micrograph (D). Tethered secretions and lumen plugs occurred foremost in anti-Fas treated airways (C,D;E). Fibrinogen immuno-reactivity was distributed in the mucus plugs exclusively in anti-Fas treated airways (E).

The airway epithelium was grossly changed after anti-Fas mAb treatment exhibiting injury with an abnormally loose structure (Figure 3A, and 3B) and containing many mucus producing cells protruding into the airway lumen. Furthermore, the mucus was being expelled into the airway lumen resulting in tethered secretions and mucus-plugs (Figure 7A–D). Immunostaining for fibrinogen showed that the mucus-plugs contained fibrinogen (Figure 7E), a marker of plasma exudation [4]. Another sign of pro-inflammatory anti-Fas mAb-induced events was a marked influx of neutrophils (Figure 4C; Table 1). The general inflammatory picture including secondary necrosis of apoptotic eosinophils remained 72 h after anti-Fas mAb treatment (data not shown). None of the above inflammatory indices was observed in animals treated with isotype control.

### Administration of anti-Fas mAb to animals without eosinophilic inflammation

To investigate whether anti-Fas treatment produced inflammation in lungs where eosinophils were absent we used four groups of animals immunized with OVA and challenged with saline. In these animals, that did not develop eosinophilic inflammation and goblet cell metaplasia, neither anti-Fas mAb treatment nor isotype control treatment induced apoptosis (at 8 h and 24 h). Importantly, neutrophilia or other pro-inflammatory signs were not detected in the saline-challenged and anti-Fas treated lung tissues.

## Discussion

This study demonstrated that anti-Fas mAb induced eosinophil apoptosis in both airway lumen and tissue. However, this treatment did not resolve the established allergic eosinophilic inflammation. Instead, of being engulfed a majority of the apoptotic tissue eosinophils underwent secondary necrosis. Additionally, the Fas receptor stimulation evoked direct cytolysis of non-apoptotic tissue eosinophils. As a result we could demonstrate that an efficient inducer of eosinophil apoptosis *in vivo*, anti-Fas mAb produced unprecedented asthma-like inflammation involving degranulation of eosinophils, increased expression of CC-chemokines, epithelial derangement, plasma exudation, neutrophilia, tethered hypersecretion, and occurrence of significant mucus-plasma plugs in mouse allergic airways. Yet, anti-Fas treatment of mice without airway eosinophilia did not evoke any sign of inflammation. The present data on airway tissue events *in vivo *contradict the current notion, based on interpretations of findings *in vitro *and in the airway lumen, that inducement of eosinophil apoptosis is a therapeutic modality in asthma.

Current allergic mouse models of asthma are characterized by eosinophilia and by airway remodeling including transformation of the epithelium into a secretory (PAS-positive) cell lining [32]. Otherwise the signs of inflammation are mild or absent, and the mouse models of asthma may be criticized for their lack of disease-like pathophysiology and histopathology [33]. It is, therefore, of interest that the present mouse lungs could exhibit 'asthma-like' exudative, eosinophilic, epithelial, and neutrophilic inflammation. After the combined allergen challenge and anti-Fas treatment the present tissue eosinophils further showed several signs of activation ranging from cytolysis of non-apoptotic eosinophils to secondary necrosis of apoptotic eosinophils. Cytolysis has not previously been observed in mouse airway tissue eosinophils [34], but is of particular interest because this mode of degranulation is prominent in human airway eosinophilic diseases [29,35]. Although the mechanisms involved in the present primary cytolysis of eosinophils are not known it is possible that Fas receptor stimulation directly induced cytolysis in these eosinophils through subcellular effects critically deviating from the apoptosis pathway [12]. The time course of cytolysis in this study with more cytolysis occurring at 8 h than at 24 h (contrary to secondary necrosis) supports the possibility that Fas directly evoked this mechanism. Fas administration in this study also evoked tethered luminal secretions and mucus plugs, as observed in asthma [36], considerably more so than observed after allergen challenge alone (this study). Moreover, as indicated by fibrinogen immunoreactivity, the present anti-Fas treatment evoked a plasma exudation process that has not previously been demonstrated in the allergic mouse models of asthma. In allergic asthma luminal levels of a large protein such as fibrinogen may better than albumin reflect the plasma exudation process, a hallmark of the disease especially at exacerbations [4]. Prior work has already established that anti-Fas antibody may evoke inflammatory responses such as hepatitis and pneumonitis [37]. Indeed, the pro-inflammatory aspect of Fas has already prompted repeated studies on the effect of this agent in the airways of allergic mice [23]. However, all previous workers, focusing largely on lumen findings, have arrived at the consensus notion that Fas-induced effects in mouse allergic airways are of an anti-inflammatory nature. The present study demonstrates the opposite. Our findings further indicate that careful studies are warranted to assess risks associated with treatment with adenovirus expressing Fas ligand. The latter construct was recently promoted as an anti-asthma treatment option [19].

Few non-eosinophilic cells became apoptotic in this study. We also detected no inflammatory response in anti-Fas-treated mice that had not developed lung eosinophilia indicating that eosinophlis were essentially involved in the present Fas-induced aggravation of allergic inflammation. Further studies are warranted to explain the present finding that both airway tissue and lumen eosinophils are particularly sensitive to Fas-receptor stimulation. The present observation that macrophages (or other cells) are not immediately engulfing the apoptotic eosinophils tallies with observations suggesting that pulmonary macrophages may be less efficient than commonly studied peritoneal macrophages as regards engulfment of apoptotic cells [38]. Reflecting an insufficient clearance, necrosis of tissue-dwelling, apoptotic eosinophils was a prominent feature in this study. Secondary necrosis of apoptotic cells is a pro-inflammatory event [39] and so is likely the primary cytolysis of eosinophils [35]. Both modes of cell death could thus be causally involved in the present aggravation of the allergic inflammation. In view of the dependence of Fas-induced inflammation on established lung eosinophilia the necrosis and the cytolysis phenomena may be major pathogenic mechanisms in this study. Also, as suggested by the present observations 3 days after anti-Fas treatment the induced inflammatory process is self-sustained continuing for a considerable length of time even in the absence of further airway provocations.

If cell clearance through apoptosis and engulfment are not working well in the lung other modes for non-inflammatory resolution of lung eosinophilia are needed. In fact, it was recently proposed that transepithelial cell egression may efficiently, and without affecting the integrity of the epithelial lining, resolve an established airway tissue eosinophilia [22]. Egression into the airway lumen clearly occurred in this study as evidenced by a developing BAL fluid eosinophilia in allergen challenged animals. The occurrence of eosinophil apoptosis in the present Fas-exposed airway tissue must have reduced the egression simply because dying cells cannot migrate. Inferentially, failure of tissue eosinophils to egress into the airway lumen (due to their apoptotic condition) explains in part the present maintained tissue eosinophilia. A reduced egression would also explain in part the demonstration in this and previous studies [7] of anti-Fas mAb-induced diminution of lumen eosinophilia in allergic airway inflammation. The possibility of such complex relationships between cell numbers and phenotypes of airway lumen and tissue, respectively, underscores the difficulty in drawing conclusions merely based on airway lumen data. Similarly, it is puzzling that mouse eosinophils exhibit piecemeal dagranulation in the airway lumen [40] but not in the airway tissue [41,42].

The present anti-Fas-induced inflammation was probably associated with *de novo *recruitment of eosinophils because the expression of several eosinophil-recruiting chemokines [43], including eotaxin, and MIP-1α were markedly up-regulated. The present non-specific increase in CC-chemokines may in part be due to an unrestricted release of bioactive proteins from eosinophils undergoing cytolysis or secondary necrosis in airway tissues. Besides eosinophilia increased airway neutrophilia is common especially in severe asthma [44]. The increased presence of neutrophils is therefore an interesting feature of the present anti-Fas treated airways. Fas itself may act as a chemoattractant for neutrophils [45] but neutrophilia was not detected in the present control group challenged with saline and receiving anti-Fas treatment. It has been suggested that tissue-toxic agents from neutrophils as well as eosinophils have a causative role in epithelial derangement. Epithelial cell loss alone may also evoke significant local neutrophilia *in vivo *[28]. These aspects indicate that neutrophilia is an expected component in the present airways where degranulated eosinophils and epithelial derangement were prominent features.

## Conclusion

This study demonstrates for the first time that eosinophil apoptosis can be induced to a great extent in airway tissues *in vivo*. But then, in contrast to current notions, this response was not associated with resolution of the established eosinophilic inflammation. This finding alone questions the proposed role of apoptosis for efficient, non-inflammatory clearance of a major granulocyte in allergic airways. Yet, we additionally discovered here that the combined effect of allergic eosinophilic inflammation and anti-Fas evoked not only apoptosis but also secondary necrosis as well as primary cytolysis of the tissue-dwelling eosinophils. These latter effects were associated with multifaceted inflammatory processes developing far beyond the allergic inflammation in this species and involving significant components of hallmark features of asthma. Hence, Fas-receptor stimulation of airway tissue eosinophils *in vivo *emerges as a significant pathogenetic mechanism and unravels significant risks involved in drug-induced stimulation of death-receptors.

## Competing interests

The author(s) declare that they have no competing interests.

## Authors' contributions

LU participated in the design of the study and played a major role in acquisition, analysis and interpretation of data and drafted the manuscript. KRT performed the statistical analysis and participated in drawing the figures. CGAP contributed to the design of the study and interpretation of *in vivo *data and writing the manuscript. JSE participated in the design of the study and helped to perform the *in vivo *procedures. All authors read and approved the final manuscript.
